# Recent Development of Radiofluorination of Boron Agents for Boron Neutron Capture Therapy of Tumor: Creation of ^18^F-Labeled C-F and B-F Linkages

**DOI:** 10.3390/ph16010093

**Published:** 2023-01-09

**Authors:** Jin-Pei Deng, Chung-Shan Yu

**Affiliations:** 1 Department of Chemistry, Tamkang University, Tamsui, New Taipei City 251301, Taiwan; 2 Department of Biomedical Engineering and Environmental Sciences, National Tsinghua University, Hsinchu 30013, Taiwan; 3 Institute of Nuclear Engineering and Science, National Tsinghua University, Hsinchu 30013, Taiwan

**Keywords:** BNCT, brain tumor, tyrosine, boronophenyl alanine, BPA, FBPA, F-18

## Abstract

Boron neutron capture therapy (BNCT) is a binary therapeutic technique employing a boron agent to be delivered to the tumor site followed by the irradiation of neutrons. Biofunctional molecules/nanoparticles labeled with F-18 can provide an initial pharmacokinetic profile of patients to guide the subsequent treatment planning procedure of BNCT. Borono phenylalanine (BPA), recognized by the *l*-type amino acid transporter, can cross the blood-brain barrier and be accumulated in gliomas. The radiofluoro BNCT agents are reviewed by considering (1) less cytotoxicity, (2) diagnosing and therapeutic purposes, (3) aqueous solubility and extraction route, as well as (4), the trifluoroborate effect. A trifluoroborate-containing amino acid such as fluoroboronotyrosine (FBY) represents an example with both functionalities of imaging and therapeutics. Comparing with the insignificant cytotoxicity of clinical BPA with IC_50_ > 500 μM, FBY also shows minute toxicity with IC_50_ > 500 μM. [^18^F]FBY is a potential diagnostic agent for its tumor to normal accumulation (T/N) ratio, which ranges from 2.3 to 24.5 from positron emission tomography, whereas the T/N ratio of FBPA is greater than 2.5. Additionally, in serving as a BNCT therapeutic agent, the boron concentration of FBY accumulated in gliomas remains uncertain. The solubility of 3-BPA is better than that of BPA, as evidenced by the cerebral dose of 3.4%ID/g vs. 2.2%ID/g, respectively. While the extraction route of *d*-BPA differs from that of BPA, an impressive T/N ratio of 6.9 vs. 1.5 is noted. [^18^F]FBPA, the most common clinical boron agent, facilitates the application of BPA in clinical BNCT. In addition to [^18^F]FBY, [^18^F] trifluoroborated nucleoside analog obtained through 1,3-dipolar cycloaddition shows marked tumoral uptake of 1.5%ID/g. Other examples using electrophilic and nucleophilic fluorination on the boron compounds are also reviewed, including diboronopinacolone phenylalanine and nonsteroidal anti-inflammatory agents.

## 1. Introduction

Fluorine-containing small molecule pharmaceuticals have impacted the drug market in the past decades [1,2]. In 2019, 13 new fluoro-pharmaceuticals were approved by the FDA, accounting for 41% of all small-molecule drugs [3]. In view of its high electronegativity as the second-smallest atom after hydrogen, its ability to form hydrogen bonds, the effect on bond strength, and its role as a conformational moderator, fluorine introduction is an efficient approach in tuning the final drug candidates to facilitate the drug discovery process [4,5]. However, fluoro substituents can act as pharmacophore isosteres, such as CF_3_,to mimic the C=O oxygen group [4]. On the other hand, fluorine compounds can act as diagnostic agents for positron emission tomography (PET) applications [6,7,8]. Its unstable isotope, the radioactive ^18^F atom, serves as the tool to extract the pharmacokinetic information. This is largely reliant on its unique internal calibrating ability, which is achieved through the emission of two 180° coherent annihilation radiations derived from each event of a radiated positron colliding with the neighboring electron of tissue. Thus, the unique physical feature enables its quantitative access to the distribution profile of the ^18^F-labeled compound in vivo, regardless of the tissue depth. The imaging data can be reformulated as an expression of counting data over volume, equivalent to a concentration level that can be compared with the initial injection concentration of drugs. The percentage of injection dose per weight of tissue (%ID/g) accumulated in organs of interest would provide straightforwardly the kinetics information of the drug. For example, as one of the most effective PET imaging agents for diagnosing cancers, [^18^F]FDG has established a gold standard for detecting a variety of tumors, such as lung cancer, head and neck cancer, brain cancer, and pancreatic cancer [9,10].

Additionally, a number of fluoropharmaceuticals were radiolabeled with F-18 to evaluate their pharmacokinetic profiles, e.g., [^18^F]F-DOPA [11,12] and [^18^F]F-MISO [13,14]. Equally important is the evaluability of the pharmacokinetics of those potential compound candidates that were readily developed from bench synthesis. Hence, the tool provides a shortcut to evaluating the in vivo performance of a compound for preclinical application.

The two principal radiofluorination sources include nucleophilic fluoride [^18^F^-^] and radiofluorine gas [^18^F]F_2_ [15,16,17,18]. In view of its short half-life (t_1/2_ =110 min), the radiofluorination is better arranged at the last step in the whole synthesis. Whereas there are abundant papers reviewing the status of fluorine- and radiofluorine-containing pharmaceuticals, we are interested in presenting our aspect of their application in facilitating the development of borono pharmaceuticals for boron neutron capture therapy (BNCT). This review will be specifically focused on the BNCT agents against brain tumors.

BNCT is a binary therapy combining a nonradioactive ^10^B atom with a beam line consisting of neutrons [19,20,21,22,23]. As being nonradioactive such as ^11^B (80%), the 20% abundant ^10^B is bombarded by neutron beams that are speeded up to an energy level of epithermal range via an accelerator or a linear reactor to reach a 10-cm penetrable depth within tissues. With a significant large cross-section σ in 3840 barns, ^10^B can capture neutrons to split highly energetic alpha and Li ions via ^10^B (n, α)^7^Li. The energy damages hereditary substances such as DNA double strands, thereby resulting in cancerous lethality.

Furthermore, to deliver the potential boron bomb towards the tumor lesions, a targetable molecule/nanoparticle or a biologically recognizable molecule/nanoparticle needs to be introduced. It has been reported that a concentration of ^10^B at 2 mM (10^9^ atoms/cell or 20–50 μg of ^10^B/g) is required to sustain a satisfactory BNCT [20,24]. Thus, determination of the concentration of ^10^B in vivo is a prerequisite to conducting a successful clinical BNCT. Not only the concentration level in the tumor lesions but also the cancerous selectivity of ^10^B needs to be taken into consideration. whereas a dividing line of 3:1 for the tumor to normal (T/N) accumulating ratio is suggested [25]. Patients with a T/N ratio as low as 2.1 are recruited, and tumor responses are obtained as expected, according to the Taiwanese clinical experience of head and neck cancers or meningiomas [26].

**Figure 1:**
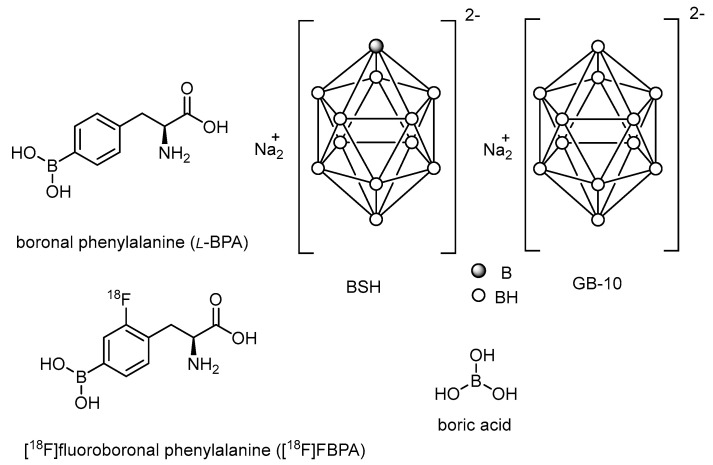
Most recently developed boron carriers for BNCT.

Of the boron-containing agents reported so far, boronophenylalanine (BPA) and borocaptate sodium (BSH) are the two most commonly used boron agents for clinical treatment (Figure 1). In addition, various boron agents have been developed in the past decades, including peptides [27,28], antibody-based delivery systems [29,30], boron compound conjugates [31,32], boron-containing nanoparticles [33,34,35,36,37,38,39,40], boric acid [41,42,43], and decahydrodecaborate (GB-10) [44,45].

## 2. Tyrosine Analogs as Boron Delivery Agents

The partially essential amino acid tyrosine crosses the blood-brain barrier through the *l*-type amino acid transporter [46,47]. Tumors, especially brain tumors such as gliomas, use tyrosine as one of the essential nutritional sources [47,48,49,50]. The typical nutritional source, such as glucose, is internalized through the glucose transporter in the brain [51]. [^18^F]FDG is such an example and is still the most successful agent for tumor imaging because of its extraordinary hunger for glucose [50]. This would explain why BPA, the structural isostere of tyrosine, has been so commonly applied in BNCT [52]. As BPA is the most clinically studied boron delivery vehicle, the boron concentration to be determined is mainly through noninvasive imaging techniques such as PET [53,54,55]. Among the most encountered cancer types, head and neck and brain tumors are difficult to resect for biopsy sampling, thus highlighting the precious value of [^18^F]FBPA [54,56].

**Scheme 1:**
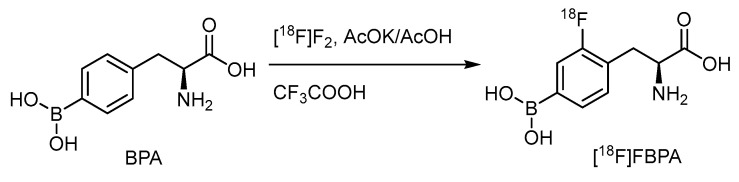
Electrophilic radiofluorination of BPA.

**Scheme 2:**
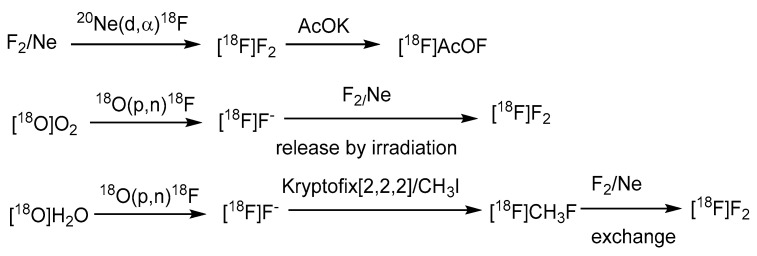
The common generation of radiofluorine source [^18^F]F_2_ for electrophilic fluorination [52]. In courtesy of the permission of copyright by publisher of Springer Nature.

Electrophilic radiofluorination on the meta position of the borono acid substituted aromatic ring produces the radiofluorinated BPA, [^18^F]FBPA (Scheme 1) [57]. BPA is one of the substrates for the LAT-1-dependent transporter, indicating the main accumulation route in gliomas. Several targets to be bombarded by accelerated particles, including the gaseous Ne (d,α) or O_2_ (p,n) and the aqueous [^18^O]H_2_O (p,n), have produced radiofluorine gas (Scheme 2). Due to the adsorption on the targeting tube, the recovery of radiofluorine through exchange with F_2_ carrier gas reduces the specific activity of [^18^F]F_2_ [52]. Whereas as less as 1/100-1/1000 times specific activity than that of the conventional radiofluoride source (GBq/mmol vs. GBq/umol), fluorine gas-derived boron compounds through the carrier-added fluorine gas better mimic the concentration of ppm level of the therapeutic dosage for BNCT. Hence, [^18^F]FBPA obtained through electrophilic fluorination is quite suitable as a theranostic agent (Scheme 2).

The radiochemical yield of [^18^F]FBPA has been optimized by adjusting the reaction conditions, e.g., pre-irradiation before [^18^F]F_2_ production, concentration of the carrier F_2_ in the Ne target, an adequate ratio of BPA to F_2_, appropriate eluents for separation through high-performance liquid chromatography (HPLC), and enantiomeric purity [57].

**Scheme 3:**
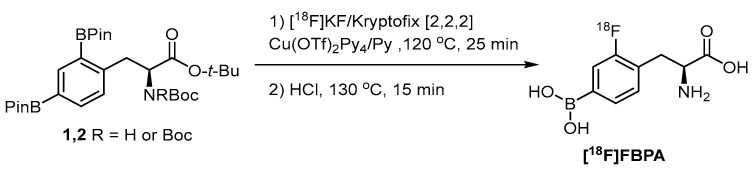
Monoradiofluorination of a diboronotyrosine compound to deliver [^18^F]FBPA [58]. In courtesy of the permission of copyright by the publisher of RSC.

Although the nucleophilic fluorination is incapable of incorporating into a borono compound due to the undesired fluorodeboronation, selective fluorination of one of the diborono groups is a straightforward approach to preserve both the imaging radiofluoro and therapeutic borono groups (Scheme 3) [58]. The copper triflate-mediated fluorination cloned FBPA with high specific activity.

Whereas the structural difference between BPA and [^18^F]FBPA is minute, variation may arise due to the different injection modes resulting in differential concentration levels [54,59]. For example, in contrast to the imaging dosage that requires only several submicromolarities of [^18^F]FBPA, therapeutic BNCT needs at least several hundred milligrams of BPA per kilogram of body weight [60,61]. The injection methods may also vary, i.e., bolus injection of [^18^F]FBPA for imaging purposes and intravenous continuous injection of BPA for BNCT. Additionally, reports have shown comparable results [54,60]. The tumoral heterogeneity and vascularity render the PET imaging inconsistent, thereby lowering the T/N ratio. According to Lo YW et al., T/N greater than 2 is accepted for clinical purposes [62]. Personalized PET imaging of [^18^F]FBPA to acquire a T/N value and the pharmacokinetics derived thereafter may contribute to a precise treatment planning procedure for BNCT [63].

**Figure 2:**
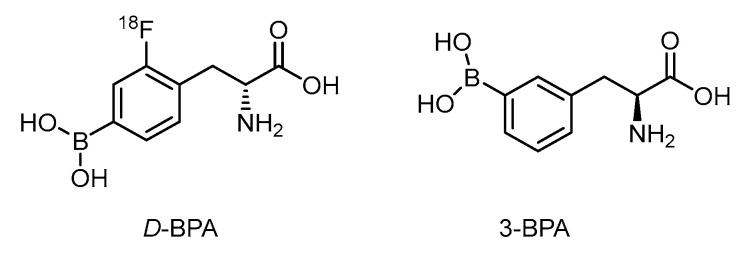
Structurally related BPA analogs as potential BNCT agents.

The *d*-isomer of FBPA is thought to be superior to the *l*-form on account of its high tumor-to-blood (T/B) ratio of 6.93 vs. 1.45 in the rat glioma model (Figure 2) [64]. The fast washout of *d*-form improves the contrast ratio. A distinct metabolism of *d*-FBPA features its major excretion route through the kidney compared with that via the liver for FBPA, however [65]. In spite of the above advantage taken by *d*-FBPA, data on boron concentration in the glioma lesion is not yet known.

**Scheme 4:**
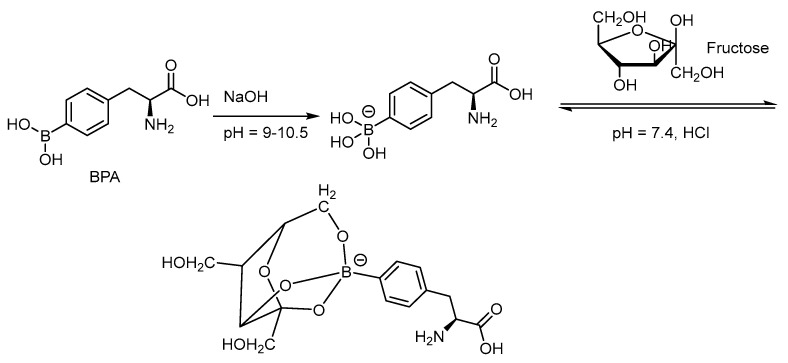
Formation of the aqueous solvable BPA-fructose complex through dehydration from the trihydroxyborate and hydroxyl groups of the furanose [66]. In courtesy of the permission of copyright by the publisher of Elsevier.

Due to the limited aqueous solubility of BPA, complexation with fructose through dehydration can shift the equilibrium to the more soluble intermediate (Scheme 4) [66]. The 3-borono isomer of BPA has shown to improve the solubility in aqueous solution 100 times better than that of the BPA-fructose complex through conformational assistance (Figure 2) [67]. 3-BPA also distributes better in the glioma of the mouse model than that of 4-BPA as reported from ^11^B ICP-MS analysis i.e., 3.4%ID/g vs. 2.2%ID/g at 2 h post injection. Until now, no F-18 tagged 3-BPA has been synthesized for PET analysis. The kinetics through PET imaging are useful to answer the metabolic route.

## 3. Other Non-Glioma-Directed Boron Agents for Radiofluorination

**Scheme 5:**
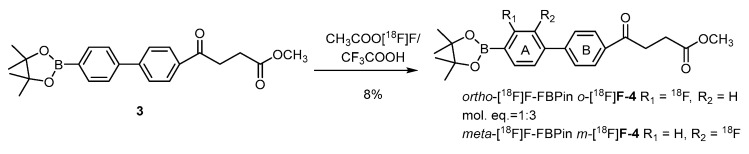
Electrophilic radiofluorination of a boronofenbufen for targeting COX-2 overexpressed cholangio carcinoma tumors [68]. In courtesy of the permission of copyright by the publisher of Elsevier.

Direct fluorination of the borono compounds is also exemplified by boronofenbufen, a COX-2 inhibitor and a member of the class of nonsteroidal anti-inflammatory drugs (NSAIDs) (Scheme 5) [68]. Cholangiocarcinoma (CCA) that overexpresses COX-2 is a fatal liver cancer with a very low cure rate. CCA is difficult to diagnose due to the foci deep inside the liver lobes. Electrophilic fluorination of fenbufen delivers the meta-radiofluoro borono fenbufen (*m*-[^18^F]FFBPin) and the ortho isomer *o*-[^18^F]FFBPin in satisfactory radiochemical yields of 6% and 2%, respectively. The fair PET-derived T/N ratio of 1.5 renders it inappropriate as an imaging agent for future BNCT usage.

**Scheme 6:**
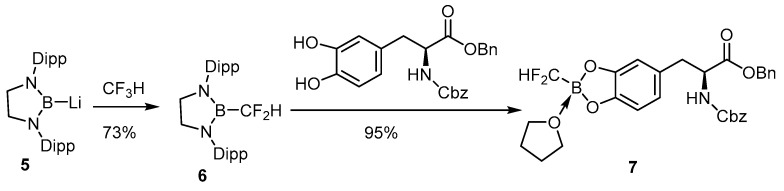
Conjugation of a boronodifluoromethylene compound to a tyrosine analog [69]. In courtesy of the permission of copyright by the publisher of JOHN WILEY AND SONS.

Insertion of the difluoromethyl group into lithium 1,3-bis(2,6-diisopropylphenyl)-1,3,2 diazaborolidinyl-2-uide activates the C-F linkage that can further react with the vicinal dihydroxy phenylalanine derivative to provide the air-stable difluoromethylborono compounds (Scheme 6) [69]. The potential difluoromethylboron tyrosine has not yet been radiofluorinated for in vivo study.

## 4. Facial Radiofluorination through Exchanging ^18^F for ^19^F on a Trifluoroborate

**Figure 3:**
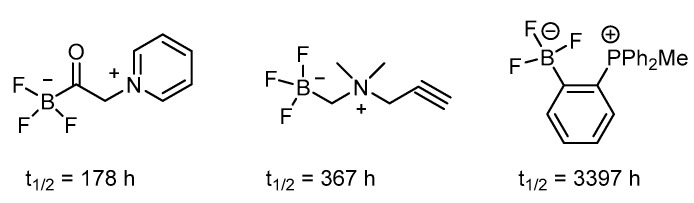
Trifluoroborates showing stability via electron-withdrawing contribution on an arene ring [70].

Heteroatomic fluoride exerts a distinct profile from carbon fluoride with respect to bond strength and radiofuorinating ability [70]. Among which, the trifluoroborate emerges as the most promising theranostic boron carrier because of its available functionality for radiofluorination via a facile radiofluoro-fluoro exchange (Figure 3) [70].

**Scheme 7:**
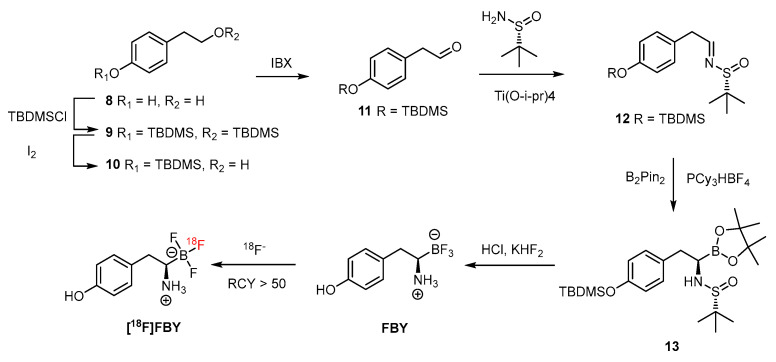
FBY and [^18^F]FBY generated through a facial synthetic route comprising a step of HPLC purification [71]. With the permission of the publisher of ACS publications.

The trifluoroborate could be generated by mixing the precursor, such as boronic acid or boronic ester, with KHF_2_ under an acidic condition of HCl. The subsequent radiofluorine exchange reaction then provides the target F-18-labeled compound in an efficient way under mild conditions. The reported radiochemical yield of greater than 50% within 15 min demonstrated its usefulness [71]. Additionally, the trifluoroborate group has been introduced to both the aromatic ring and aliphatic chain. The stability of aryl trifluoroborate is dependent on the substituent and is mostly stabilized when the electron-withdrawing group is in the ortho or para position. Further, the presence of the trialkylphosphonium salt can prolong the half-life to 3397 h (Figure 3). The zwitter ion-like nature of an amino acid is retained when the carboxyl group is replaced by the tifluoroboron such as in FBY (Scheme 7) [71].

As reported by Li et al., the preparation is facile before the step of introduction of boronopinacolone in the presence of PCy3·HBF_4_ [71]. After a sophisticated purification with semipreparative HPLC, a relatively low yield (30%) of FBY was obtained. The FBY has shown remarkable stability, with 98% intact form after treatment with H_2_O_2_ solution for 4 h, compared with the 99% conversion in 1 h by BPA. A human study by radiofluoro [^18^F]FBY has shown a high T/N ratio of 2.30 ± 1.26 and 24.56 ± 6.32 in low- and high-grade tumors, respectively [72]. Whereas the concentration of boron in human studies is not available from [^18^F]FBY, a concentration of 19.59 ppm was reported for melanoma tumors of xenografted mice [71,73]. As a diagnosing agent, the T/N radioactivity ratio is a decisive index, and FBY has shown to be a promising PET imaging agent for diagnosing tumors. For use as a therapeutic agent against tumors, boron concentrations must be as high as possible. In comparison with its high tumoral accumulation [72,74], the uptake of <2%ID/g in brain tissue of the mouse model seems to be surpassed by that of the glioma model in the clinical study. In addition, the preparation of target FBY might constitute a bottom neck in future applications of this BNCT agent because a glioma patient requires an injection dose of BPA of 450 mg/kg equivalent to 32 g for a 70 kg patient, for each irradiation. The aqueous solubility is also needed to be considered. For example, the insufficient solubility of BPA can be improved by complexing with the trihydroxy groups of fructose via condensation. Without auxiliary groups, FBY needs to be water dissolvable through its inherent properties. Nevertheless, the excellent stability of FBY might compensate for the tumoral boron concentration.

**Figure 4:**
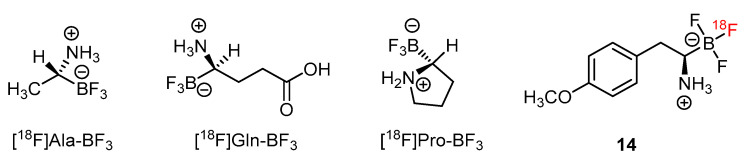
Radiofluoride-exchanging trifluoroborate-substituted amino acid analogs for tumor imaging [75].

In addition to [^18^F]FBY, a number of amino acids have been tagged by radiofluoride through the exchange reaction on the trifluoroborate moieties [75]. Examples such as [^18^F]Ala-BF_3_, [^18^F]Gln-BF_3_ and [^18^F]Pro-BF_3_ showed their values as future BNCT theranostic agents (Figure 4).

**Scheme 8:**
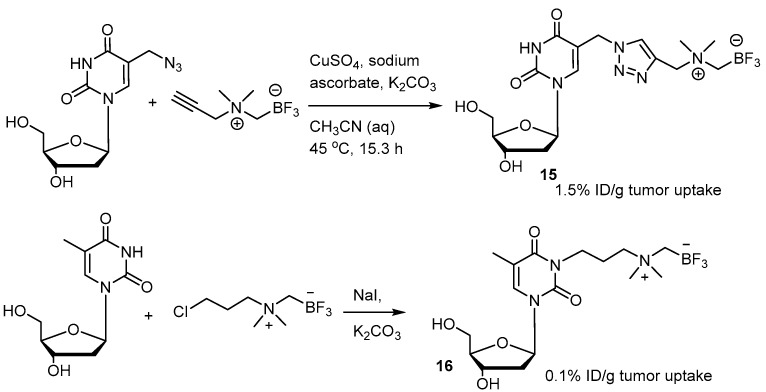
Nucleoside trifluoroborate **15** formed from 1,3-dipolar cycloaddition compared with the *n*-alkylated trifluoroborate nucleoside **16** in tumoral uptake [76]. In courtesy of the permission of copyright by publisher of RSC.

A series of derivative [^18^F]FBY analogs were developed for studying the uptake of radioactivity in tumors of B16-F10 xenograft mice [73]. Among them, the *p*-methoxy derivative **14** exerted a tumoral uptake of 3.5%ID/g (Figure 4). 1,3-Dipolar cycloaddition, also known as click chemistry, has been used to combine the trifluoroborate moiety with the nucleoside to give the zwitter ionic nucleoside (Scheme 8) [76]. A tumoral uptake value of 1.5%ID/g for **15** in an U87 Xenograft mouse is much better than that of the *n*-alkylated trifluoroborated nucleoside, 0.1%ID/g. The uptake value of the brain is not provided.

## 5. Conclusions

### 5.1. Less Toxicity

Unlike the chemotherapeutic drugs required to be highly cytotoxic against tumors concurrently with minor side effects, BNCT agents merely need to be taken by the tumor in a concentration as high as possible without significant cytotoxicity. Hence, it may be expected that the hit rate of potential clinical boron compounds would be higher than that of the chemotherapeutic agents.

### 5.2. Diagnostic Purpose and BNCT Therapeutic Purpose

According to the viewpoint of diagnosing gliomas, [^18^F]FBY is better than [^18^F]FBPA with respect to the high T/N ratio, ranging from 2.3 to 24.5 vs. greater than 2.5, respectively. In serving as the BNCT therapeutic agent, the concentration of boron in the glioma of FBY has not yet been provided, except for a xenografted melanoma with a concentration of 19.6 ppm. This value may be hampered by the limited cerebral accumulation dose of its radiofluorocongener at 0.4%ID/g. Moreover, subtly released radiofluoride was accumulated on the skull of a glioma patient, implying an exchange of fluoride for the surrounding anion. Thus, compared with BPA, which can reach a concentration of less than 10 ppm and increases to 20–40 ppm through BPA-fructose conjugate coupled with intravenous continuous injection, FBY needs more evidence to support enough boron in gliomas.

### 5.3. Extraction Route and Solubility

In spite of the fact that *d*-BPA shows a better T/N of 6.9 than that of BPA, which is 1.5, further animal studies are required because the extraction route is solely through the kidney rather than the partial liver, as is the case with BPA. The 3-BPA has better aqueous solubility than that of BPA, as reflected by the cerebral dose of 3.4%ID/g vs. 2.2%ID/g. Whereas radiofluorinated 3-BPA through electrophilic fluorination remains an obstacle at the present stage, a nucleophilic pathway may be available in the future, thereby enabling the determination of the T/N ratio.

### 5.4. Trifluoroborate Effect

The fair tumoral uptake of trifluoborate-conjugated natural products such as nucleoside derivatives (1.5%ID/g) is noted. Radiofluorination enables their straightforward assessment of the T/N ratio and boron concentration in vivo.

In spite of the unsatisfactory tumoral uptake and T/N selectivity, BPA remains the most commonly used clinical BNCT agent. PET imaging of F-18-labeled FBPA presents the personalized kinetics of BPA to guide the BNCT treatment planning procedure. Additionally, not only having both characteristics of imaging and therapeutics in a functional group, F-18-labeled trifluoroborate can adequately mimic the COOH group of an amino acid. [^18^F]FBY appears to be the potential imaging agent for glioma, thus encouraging its nonradioactive FBY to enroll in the clinical trial for BNCT.

Radiofluorination with F-18 is still impacting the development of BNCT agents and other therapeutic agents. As indicated by the roles played by radiofluoroborono compounds in BNCT research, more potentially clinically useful BNCT agents may emerge in due course. The F-18 labeling facilitates the preclinical assessment of those borono compounds just delivered from bench work. The most crucial contribution of F-18 is that an organic chemist can perform a biological assay beyond a cell culture study to save time on the development of boron compounds to meet the features required by metabolic stability, aqueous solubility for injection formulation, lesion targeting ability, and dosage calculation.

## Data Availability

Not applicable.
